# Black Mulberries (*Morus nigra* L.) Modulate Oxidative Stress and Beta-Amyloid-Induced Toxicity, Becoming a Potential Neuroprotective Functional Food

**DOI:** 10.3390/foods13162577

**Published:** 2024-08-17

**Authors:** Guillermo Cásedas, Cristina Moliner, Alba Abad-Longas, Sonia Núñez, Carlota Gómez-Rincón, Filippo Maggi, Víctor López

**Affiliations:** 1 Department of Pharmacy, Faculty of Health Sciences, Universidad San Jorge, 50830 Zaragoza, Spain; gcasedas@usj.es (G.C.); acmoliner@usj.es (C.M.); abad@usj.es (A.A.-L.); snunez@usj.es (S.N.); cgomez@usj.es (C.G.-R.); 2 Instituto Agroalimentario de Aragón-IA2, CITA-Universidad de Zaragoza, 50009 Zaragoza, Spain; 3 Chemistry Interdisciplinary Project (ChIP) Research Center, School of Pharmacy, University of Camerino, 62032 Camerino, Italy; filippo.maggi@unicam.it; 4 Facultad de Ciencias de la Salud, Universidad San Jorge, Campus Universitario Villanueva de Gállego, Autovía A-23 Zaragoza-Huesca Km. 299, 50830 Zaragoza, Spain

**Keywords:** black mulberry, phenolic compounds, antioxidant activity, ROS, neuroprotection, functional foods

## Abstract

Black mulberry (*Morus nigra* L.) is a common edible fruit from the Moraceae family with a wide variety of nutritional and medicinal applications, mainly due to its antioxidant and anti-inflammatory properties. The purpose of this work was to investigate the cytoprotective and neuroprotective capacity of a hydrophilic black mulberry solvent-free extract rich in polyphenols, including the antioxidant, antiradical, and enzymatic mechanisms that would explain these effects. Its neuroprotective potential was evaluated in vitro using the Neuro-2a cell line and in vivo through the *Caenorhabditis elegans* organism model. Neuro-2a cells were treated at different concentrations of the extract (25–500 µg/mL) and hydrogen peroxide (300 µM) as an oxidant agent, simultaneously. From these treatments, redox status (intracellular ROS production) and cellular activity (MTT) were also quantified in Neuro-2a. Regarding the *C. elegans* assay, the protection of the extract against β-amyloid toxicity was measured against the CL4176 strain, which is a model of Alzheimer disease. As a complementary neuroprotective assay, its potential to inhibit the monoamine oxidase A (MAO-A) enzyme was measured. In addition, an *Artemia salina* bioassay was performed for preliminary toxicity screening. And its antioxidant properties were evaluated by means of the FRAP assay. The results confirm its neuroprotective potential and its ability to scavenge free radicals and decrease ROS production, also acting as a moderate MAO-A inhibitor. Moreover, the polyphenolic extract alleviates the toxicity induced by β-amyloid accumulation in *C. elegans*. Concluding, *Morus nigra* can be considered a functional food with bioactive compounds that may prevent the onset of neurodegenerative diseases.

## 1. Introduction

The interest in and demand for the consumption of fresh fruits has currently increased among the population. This can be attributed to greater awareness of healthy habits on the part of consumers, with the berry being a powerful source of antioxidant properties [1].

**Figure 1:**
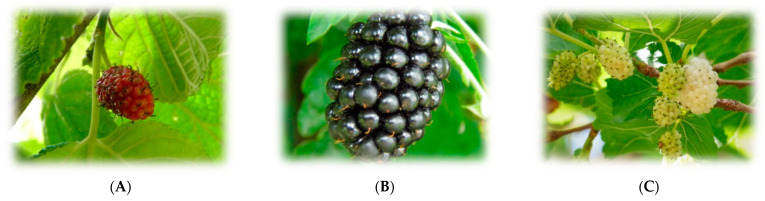
Most common Morus species. (**A**) *Morus rubra*. (**B**) *Morus nigra*. (**C**) *Morus alba*.

The black mulberry, *Morus nigra* L., belongs to the Moraceae family. There are 24 species of *Morus* and one subspecies, with at least 100 known varieties. We can find the black mulberry distributed in a wide variety of areas, with the ability to grow in a wide range of topographic, climatic, and soil conditions [2]. Within the genus *Morus*, there are three main species for fruit production (Figure 1): red (*M. rubra* L.), black (*M. nigra*) and white (*M. alba* L.) mulberry [3]. *M. nigra* and *M. alba* have fruits that are 2–3 centimetres long and are green in their immature phases. *M. nigra* acquires a purple-black colour at maturity due to its high amount of flavonoids and anthocyanins [4,5]. *M. alba* reaches a pinkish white colour in its mature state, due to its low concentrations of flavonoids and anthocyanins, presenting a sweet and insignificant flavour [6]. *M. rubra*, on the other hand, acquires a red colour throughout its maturity; along with *Morus nigra*, they are the ones that provide the most acidity due to their low pH (Ercisli & Orhan, 2007). The black mulberry is mostly consumed directly from the fruit itself; among its wide variety of applications, it can be found in the form of syrups, jams, vinegar, or alcoholic beverages made from it [7].

*M. nigra*, in addition to being a food, has been used in traditional medicine for thousands of years, mainly in China, to treat sore throat, anemia, and tonsillitis [8]. It has been shown to have a wide range of beneficial properties including anti-inflammatory, antidiabetic, antinociceptive, antimicrobial, anti-melanogenic (involved in skin whitening), anticancer, antihyperlipidemic, and anti-atherosclerotic activities [9,10,11,12,13,14]. It has also shown therapeutic and protective effects on the central nervous system, kidneys, liver, gastrointestinal tract, and female reproductive system [15,16,17,18]. All of this suggests that black mulberry is a very good resource for controlling and preventing a wide range of chronic diseases [19].

This species is known for its slightly acidic flavour and high nutritional value, containing a high amount of fibre and a good dose of iron, calcium, and potassium [7], in addition to being an excellent source of the numerous phytochemical components to which we attribute most of these characteristics, mainly due to their antioxidant capacity. The main bioactive compounds present in black mulberry are phenolic acids, flavonoids, and anthocyanins. The anthocyanin content is significantly higher than in red or white mulberries [20].

Polyphenols, better named as phenolic compounds, have been considered one of the most important group of secondary metabolites with bioactive properties in the plant kingdom. They have an aromatic ring and contain at least one phenol group in their molecular structure [21]. It has been shown that a diet rich in fruits containing phenolic compounds could prevent oxidative damage caused by ageing and certain diseases (such as Parkinson’s disease, multiple sclerosis or cancer) [22], responsible for eliminating free radicals from metabolism cells. It is known that they are responsible for participating in protection against the harmful actions of reactive oxygen species (ROS) [23].

Phenolic compounds are a large family classified by their structural characteristics as flavonoids (flavones, flavanones, flavonols, isoflavones, catechins, anthocyanins), stilbenes, coumarins, tannins, lignans, and phenolic acids (such as hydroxybenzoic and hydroxycinnamic acids) [24]. The flavonoids present in black mulberries include quercetin-3-glucoside, rutin, and quercetin-3-malonyl glucoside. Phenolic acids in black mulberry include chlorogenic, gallic, syringic, and caffeic acids [25].

On the other hand, a key component in blackberry fruits are anthocyanins, which are part of the natural phenolic compounds to which are attributed the colour properties and biological activities such as antimicrobial, antioxidant, neuroprotective, and anti-inflammatory capacities [26]. Regarding their chemical structure, they are anthocyanidin glucosides, formed by an aglycone molecule that is held together by a glycosidic bond to some sugar whose colour intensity depends on the position and number of free -OH; the higher the number, the greater the intensity of the blue that it contributes to the fruit [27]. Recently, interest in anthocyanin pigments has increased thanks to their therapeutic and pharmacological properties [28]; antidiabetic, anti-inflammatory, and antitumor effects [29]; and the improvement of cognitive behaviour and visual acuity and the inhibition of the oxidation of some lipoproteins [30]. The main anthocyanins detected in this *M. nigra* extract are cyanidin-3-O-glucoside and cyanidin-3-O-rutinoside, and as minor components, cyanidin-3-O-(6-malonyl-glucoside) and cyanidin-3-O-(6-dioxalyl-glucoside) [25].

A large number of organic acids are also found in black mulberries as water-soluble materials found in the cytoplasm, accompanied by sugars, which contribute to the flavour of the fruit. The most predominant in this species of mulberry are malic, citric, tartaric, succinic, lactic, fumaric, and acetic acids [7,31], with the content of succinic acid being greater than that of tartaric acid, in variation with the other two species. Differences may be due to genetic and ecological factors. These acids constitute a complex with metal ions, causing their oxidation to prevent the catalyst effect. They have a great impact on the flavour; it is reduced and favours the acidity of the fruit. The link between the total content of organic acids and the amount of sugars in fruits is an important criterion of ripening [7].

Seeing the rising demand for black mulberries, along with the fact that the phenolic compounds found in this fruit are beneficial to human health, the development of rapid and reliable methods to extract these chemicals is required. The most used extraction for crushed fruit is solid–liquid extraction. For good performance of the technique, the method used is very important, as well as the process factors that take into account the quantity and quality of the compound to be extracted [32]. There are a large number of extraction techniques in the literature: microwave-assisted extraction, pressurized fluids, ultrasound, etc. For this research, an innovative technique has been used: microwave-assisted hydro-diffusion and gravity extraction (MHG). This application revolutionizes solvent-free microwave extraction by utilizing water directly for extracting hydrophilic phyto-constituents from the matrix within the reactor. The purpose is to maximize all the biological activities of the black mulberry, being a very innovative technique. In addition, it is considered a green ecological and valuable technique, especially for the production of polyphenolic extracts. Studies suggest, due to its advantages in time and money, its greater use in both the pharmaceutical and food industries [25].

Epidemiological findings suggest that consumption of berries rich in anthocyanins and polyphenols may reduce the risk of neurodegenerative diseases, suppressing neurotoxic effects in cellular models [33]. The central nervous system and brain, compared to other tissues, are more vulnerable to oxidative damage due to their high oxygen consumption, high lipid content, and low concentration of antioxidant enzymes [34]. This damage plays an important role in the pathogenesis of neurodegenerative diseases, caused by the excessive accumulation of ROS, involved in many neurodegenerative processes such as Alzheimer’s disease, Parkinson’s disease, Huntington’s disease, Amyotrophic Lateral Sclerosis, and chronic inflammatory diseases or diabetes [35]. Despite this, the identification of neuroprotective agents remains a challenge.

Experiments conducted on rodents have shown a reduction in brain ageing when they are administered strawberries, blueberries, or blackberries [36]. Understanding the key benefits of polyphenols across various pathways and proteins is crucial for enhancing their effectiveness in addressing complex health issues [37]. In this way, the purpose of this work was to investigate the cytoprotective and neuroprotective capacity of black mulberry, including the antioxidant and enzymatic inhibitory mechanisms, using in vitro cell cultures and a transgenic strain of *Caenorhabditis elegans* of the Alzheimer’s disease model.

## 2. Materials and Methods

### 2.1. Reagents and Chemicals

The Neuro-2a (N2a) cell line was purchased from the American Type Culture Collection (ATCC, Manassas, VA, USA). *Artemia salina* was acquired from a local animal store (Zaragoza). Dulbecco’s Modified Eagle’s Medium (DMEM), Phosphate-Buffered Saline (PBS), Fetal Bovine Serum (FBS), penicillin–streptomycin, Trypsin, 3-(4,5-dimethylthiazol-2-yl)-2 Bromide, 5-Diphenyltetrazol (MTT), sea water, 2′,7′-dichloro-dihydrofluorescein diacetate (DCFH-DA), dimethyl sulfoxide (DMSO), glucose, HCl, hydrogen peroxide (30% *w*/*w*), glacial CH_3_CO_2_H, 2,4,6-tris(2-pyridyl)-1,3,5-triazine (TPTZ), FeCl_3_-6H_2_O, FeSO_4_-7H_2_O, KH_2_PO_4_, tyramine, vanillic acid, aminoantipyrine, horseradish peroxidase (HRP), monoamine oxidase A (MAO-A), and Clorgyline were acquired from Sigma-Aldrich (Barcelona, Spain).

### 2.2. Black Mulberries and Extraction of Bioactive Compounds

Ripe fruits of *M. nigra* were harvested from trees in Camerino, Italy (June 2020). Prof. Filippo Maggi, researcher at the University of Camerino, certified the botanical identity of the samples, deposited under the codex CAME#28448 in the *Herbarium Camerinensis*, School of Bioscience and Veterinary Medicine, University of Camerino. Until extraction, the samples were kept in a freezer at −18 °C [25]. The extraction was carried out by a new procedure, i.e., microwave hydro-diffusion and gravity extraction (MHG). A total of 500 g of fresh intact fruit was placed in a reactor and heated without the addition of any solvent or water (at atmospheric pressure). The microwaves interacted with the biological water, allowing the release of the bioactive compounds within the cells of the mulberry fruits. The raw juice was later collected, freeze-dried, and kept at −20 °C after extraction [25]. The composition of the “juice” in terms of phenolic compounds was previously published and mainly consisted of cyanidin 3-glucoside, pelargonidin-3-glucoside, rutin, hyperoside, isoquercitrin, and chlorogenic acid [25].

### 2.3. Neuroprotection Models

#### 2.3.1. Neuro-2a Cell Line

##### Cell Culture

Mouse neuroblastoma Neuro-2a (N2a) cells were thawed from a liquid nitrogen tank (37 °C bath, 2–3 min) and cultured in DMEM supplemented with 10% FBS and penicillin–streptomycin (2 mg/mL); they were seeded in a T75 flask and left in an incubator (5% CO_2_, 37 °C) for one or two weeks. Once the cells reached confluency, cell passage (6) was performed. Cell culture medium was frequently reinstated every three days. Once the passage was carried out, seeding was carried out in 96-well plates (10,000 cells per well). As a final step, the cells were incubated for 24/48 h until reaching total confluency in the well and being able to subsequently treat the plate.

##### N2a Treatments

Cells were pre-treated with lyophilized mulberry extract in a range of concentrations from 25 µg/mL to 1000 µg/mL for 24 h. Additionally, hydrogen peroxide insult (300 µM) was administered for 45 min to test neuroprotective potential. This study evaluated the protective effects at concentrations of 25–50–100–200 µg/mL, since these levels were non-toxic and reflected a physiologically significant amount of polyphenols.

##### Mitochondrial Activity by MTT Assay

After treatments, cell viability was measured by adding MTT solution (2 mg/mL) for 2 h at 37 °C. Sequentially, DMEM was aspirated and DMSO was placed in each well to dissolve formazan crystals. Finally, absorbance was read at 550 nm with a Synergy H1 Multi-Mode Reader (Biotek, Winooski, VT, USA) [38]. All experiments were performed five times and the results are expressed as percentage of control (100%).

##### Detection of Intracellular ROS

Neuro-2a cells were seeded in a 96-well plate. After 24 h, we replaced the medium with a solution of glucose, PBS, and DCFH-DA (2,7-di-chloro-dihydrofluorescein diacetate) protected from light. Each well contained 200 µL of said solution and was incubated for 30 min at 5% CO_2_ and 37 °C, protecting the plate with tin foil. After that time, PBS was removed by washing the plate twice with fresh glucose PBS. Then, 200 µL of PBS including different concentrations (25, 50, 100, and 200 µg/mL) of black mulberry (*Morus nigra*) extract, as well as hydrogen peroxide (300 μM), was administered [34].

The reading of the absorbances at 480 nm (λ excitation) and 520 nm (λ emission) started in the Synergy H1 Multi-Mode Reader fluorometer (Biotek), measuring the kinetics for 90 min (10 readings). The results were represented as a percentage of intracellular ROS production (over control).

#### 2.3.2. Caenorhabditis Elegans

##### Worm Strain and Maintenance

The *C. elegans* transgenic strain CL4176 (dvIs27[myo-3p::A-Beta (1–42)::let-851 3′UTR) + rol-6(su1006)]) was provided and *Escherichia coli* OP50 was obtained from the Caenorhabditis Genetics Centre (Minneapolis, MN, USA).

The CL4176 strain was developed to express human amyloid β_1–42_ (Aβ) by temperature upshift. The accumulation of Aβ peptides in muscles results in the progressive paralysis of these mutants. The worms were grown on Nematode Growth Medium (NGM) plates seeded with *Escherichia coli* OP50 at 16 °C.

##### Amyloid-β Peptide Toxicity: Paralysis Assay

The paralysis assay was performed according to the method described by Dostal and Link [39]. Briefly, age-synchronized CL4176 worms were cultured in NGM containing the extract or in the absence of it (control) for 38 h at 16 °C, and then the temperature was upshifted to 25 °C to induce expression. After 20 h, the paralysis was scored. Paralysis was assumed when the worm failed to complete one sinusoidal turn after being touched on the head and tail with a platinum wire. For each replicate, at least 50 worms were studied per condition.

### 2.4. Artemia Salina Safety and Toxicity Assessment

Dried *Artemia salina* cysts were incubated in seawater with aeration for one week. The black mulberry extract (*Morus nigra*) was dissolved in seawater (2 mg/mL). Then, ten *Metanauplius* were transferred to 6 plates with 6 wells each, subjected to different treatment concentrations (25, 50, 100, 200, 300, 400 and 500 µg/mL). Control wells were filled with 4 mL of seawater and ten *Metanauplius* [40]. To study the viability of brine shrimp at the *Metanauplius* stage and adult stages of their cycle, the assay was performed at the same time. Survival viability was calculated as an average of the wells after incubation at aerated room temperature for 24 h.

### 2.5. FRAP Assay: Ferric Reducing/Antioxidant Power

The FRAP test was performed to establish the ferric reducing/antioxidant power of the *Morus nigra* extract. This is based on the reduction of the iron (III) complex, 2,4,6-Tris (2-pyridyl)-s-triazine (TPTZ), to an iron (II) complex of intense blue colour [41,42,43]. The mulberry extract (1 mg/mL) was mixed with the FRAP reagent in Eppendorf tubes and then transferred into 96-well plates in triplicate (41–43). Optical density was measured at 595 nm in the Synergy H1 Multi-Mode Reader (Biotek) and the results were expressed as µmol Fe^2+^/g of extract.

### 2.6. Inhibition of MAO-A Bioassay

The MAO-A inhibition assay was performed in a 96-well plate. Sample wells contained black mulberry extract at different concentrations (0.03–1 mg/mL) dissolved in phosphate buffer (pH = 7.4), chromogenic solution (0.8 mM vanillic acid, 417 mM 4-aminoantipyrine and 2 U/mL horseradish peroxidase in phosphate-buffer solution), tyramine (0.2 M) and MAO-A (8 U/mL) [44]. Clorgyline (1 mg/mL) was used as a standard reference inhibitor, a selective inhibitor of MAO-A of irreversible type, with antidepressant activity. Control wells contained phosphate buffer instead of the extract. MAO-A enzyme was replaced by buffer in blanks. Optical density was read at 490 nm every 5 min for 30 min in the Synergy H1 Multi-Mode Reader (Biotek) plate reader. The assay was performed thrice.

### 2.7. Statistical Analysis

The experiment was conducted a minimum of three times. The data are presented as means ± SEM from all experiments conducted. Statistical analyses were performed using either the Student *t*-test or one/two-way ANOVA followed by Tukey’s post hoc test as well as nonlinear regression. Paralysis curves were analyzed by Kaplan–Meier survival curves and by conducting a log-rank test. Differences were considered significant when *p*-values < 0.05. All statistical analyses and figure preparations were conducted using GraphPad Prism v.8.

## 3. Results

### 3.1. Effect of Black Mulberry Extract on Neuro-2a Cell Viability

**Figure 2:**
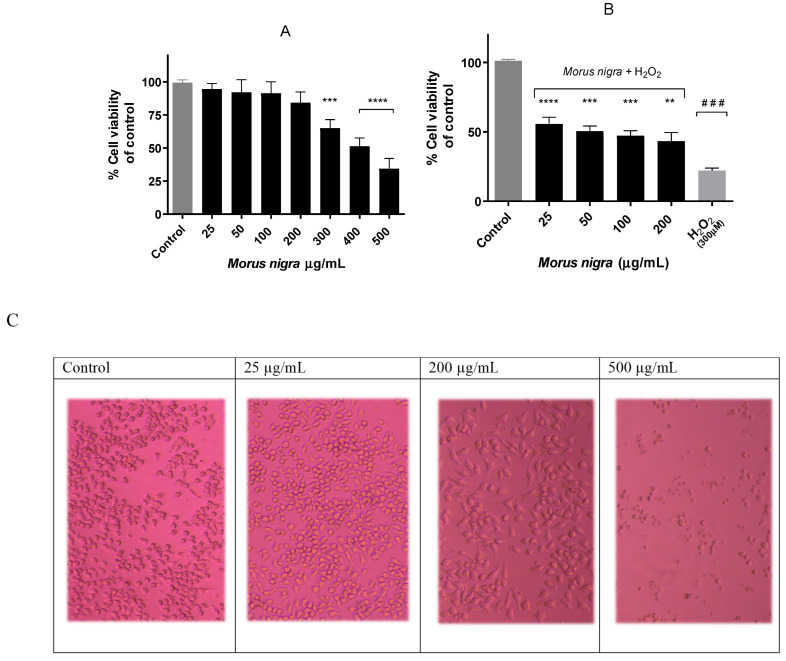
Mitochondrial activity in Neuro-2a cells (MTT assay). (**A**) Cytotoxicity in Neuro-2a cells after exposure to different concentrations of black mulberry extract (Morus nigra). Note: **** *p* < 0.0001 and *** *p* < 0.001 compared to control. Differences calculated using one-way ANOVA. (**B**) Cytoprotective effect of black mulberry extract on Neuro-2a cells against hydrogen peroxide. Note: **** *p* < 0.0001, *** *p* < 0.001 and ** *p* < 0.01 compared to hydrogen peroxide. ### *p* < 0.001 compared to control. Differences calculated using one-way ANOVA. (**C**) Microscope images of different concentrations compared to the control. Differences in toxicity of the extract (300, 400, 500 µg/mL Neuro-2a apoptosis).

Cell viability was assessed in Neuro-2a cells using the MTT assay. In this case, different concentrations of black mulberry extract (25–500 μg/mL) were tested in neurons for 24 h (Figure 2).

In Figure 2A, it can be observed how in the concentration range of 25–200 μg/mL, the viability barely decreased, but on the contrary, in the concentration range of 300–500 μg/mL, a greater decrease in cell viability with respect to the control can be seen.

To evaluate the cytoprotective capacity of *M. nigra* extract, the MTT test was performed by adding hydrogen peroxide for 45 min after 24 h extract treatment. Regarding Figure 2B, there is a slightly dose-dependent response against hydrogen peroxide; 25 μg/mL demonstrated the best cytoprotective profile compared to other concentrations. Cell viability decreased more than 75% over control cells, while pre-treated cells improved this mitochondrial response, preserving around 50% of cell viability.

### 3.2. Effect of Black Mulberry Extract Concentrations on Intracellular ROS Production in Neuro-2a Cells

To know the amount of intracellular ROS production in Neuro-2a cells, we subjected them to oxidative stress (DCFH-DA), measuring their levels for 90 min.

**Figure 3:**
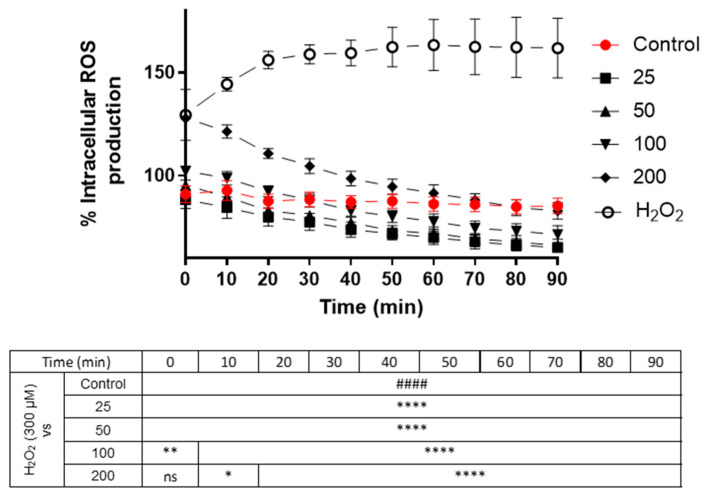
ROS production in Neuro-2a cells subjected to oxidative stress by hydrogen peroxide (300 μM) and treatment with blackberry extract (25, 50, 100 and 200 μg/mL). The data are expressed as a percentage of the control cells. Note: **** *p* < 0.0001, ** *p* < 0.01 and * *p* < 0.05 versus hydrogen peroxide. #### *p* < 0.0001 versus control; ns: not significant. Two-way ANOVA was used as statistical analysis.

As seen in Figure 3, cells treated with hydrogen peroxide (positive control) reached 160% ROS generation during the protocol, while the control cells (untreated) maintained a regular level close to 100% production. The cells treated at non-toxic concentrations (25, 50, 100 and 200 μg/mL) of the mulberry extract showed lower production even than the control during the 90 min experiment (*p* < 0.001), which means that the extract is capable of neutralizing the formation of intracellular free radicals in neurons, resulting in less than 20–25% ROS production compared to control figures.

### 3.3. Morus Nigra Extract Prevents In Vivo Aβ Toxicity in C. elegans

**Figure 4:**
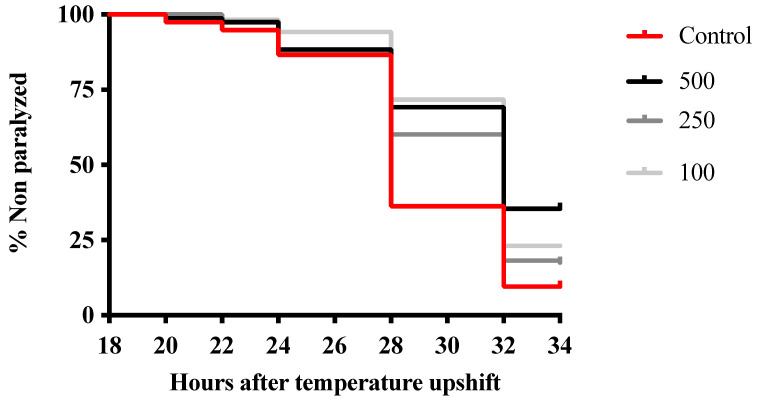
Effect of *M. nigra* extract on paralysis curves in *C. elegans* CL4176. At least 150 worms in three replicates were studied per condition. PT50 was 28 h for the control group and 32 h for worms treated with the extracts. The results of the paralysis assay were analyzed using the Kaplan–Meier survival model and for statistical significance by using a log-rank pairwise comparison test.

In order to determine the potential neuroprotection of the extract, the paralysis assay was performed using the CL4176 strain, which expresses Aβ peptide. The accumulation of Aβ peptide is neurotoxic and caused paralysis in the worms. As shown in Figure 4, all tested concentrations of the extract had a positive impact, delaying the time to become paralyzed regardless of the extract concentration. The highest concentration, 500 μg/mL, exhibited the best result, especially 32 and 34 h after the increase in temperature. The time when 50% of nematodes were paralyzed, PT50, was 28 h for the control group and 34 h for all treated groups. Therefore, the treatment increased PT50 by approximately 21%. The extract was demonstrated to alleviate the toxicity induced by Aβ overexpression in *C. elegans*.

### 3.4. Effect of Morus Nigra Extract on Artemia Salina Viability

**Figure 5:**
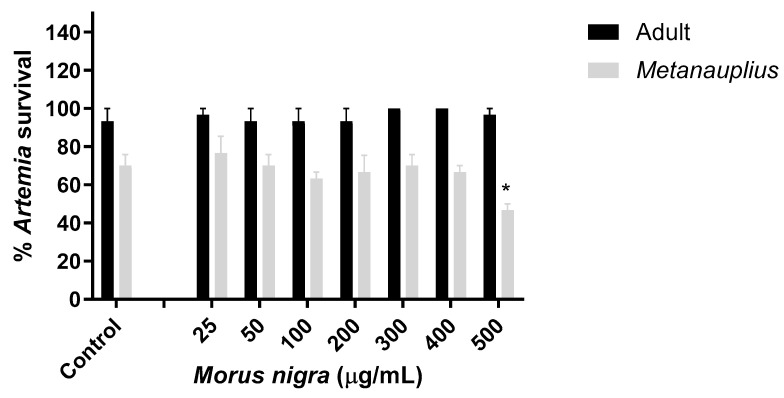
Non-toxic effect of black mulberry on *Artemia salina*. Significant differences were observed between control and 500 µg/mL treatment in *Metanauplius* stage (* *p* < 0.05). Significant differences were calculated through ANOVA and Dunnett’s Multiple Comparison Test.

This assay was carried out with the aim of evaluating the toxic capacity of the blackberry extract in *Artemia salina*, both in its *Metanauplius* stage and in its adult phase. They were exposed to different concentrations (25–500 μg/mL) compared to the control with seawater. After 24 h of treatment, survival viability was calculated. As seen in Figure 5, the *Artemia salina Metanauplius* exposed to higher concentrations had decreased (500 μg/mL) viability compared to the control, showing significant differences, while adult *Artemias* appeared intact at most concentrations, with 100% survival compared to the control. The test confirmed the anticipated outcome: the black mulberry extract showed no toxicity in this bioassay.

### 3.5. Evaluation of In Vitro Antioxidant Activity

The FRAP assay was executed with the purpose of evaluating the antioxidant activity of the black mulberry extract through radical scavenging in vitro. The average of the three readings obtained from the aliquots of the extract at a concentration of 1 mg/mL was 12.48 µmol Fe^2+^/g. The results were expressed as µmol Fe^2+^/g as the ferric reducing/antioxidant power (from iron III to iron II) is determined using an iron sulphate (FeSO_4_) standard curve.

### 3.6. Inhibitory Effect of the Extract on the MAO-A Activity

**Figure 6:**
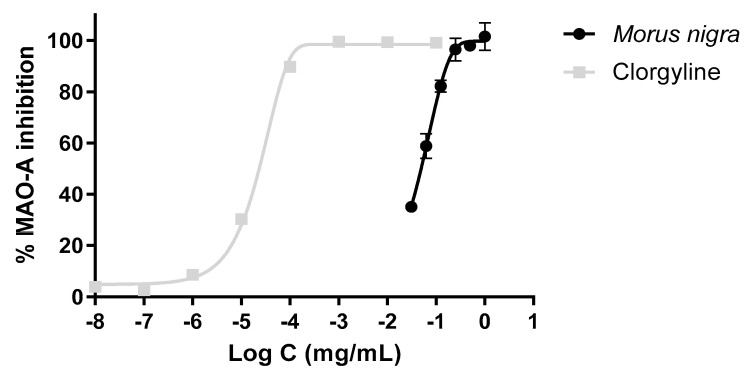
MAO−A inhibition by *Morus nigra* and Clorgyline. IC_50_ = 0.023 µg/mL and 49.64 µg/mL for Clorgyline and black mulberry extract, respectively.

The black mulberry extract showed clear enzyme inhibitory activity. IC_50_ values were calculated by nonlinear regression for the reference inhibitor (IC_50_ = 0.023 µg/mL). Figure 6 shows the profile of the *M. nigra* extract against Clorgyline, showing how both substances are capable of inhibiting MAO-A. In the case of Clorgyline (reference inhibitor), its IC_50_ value is much lower than that of the black mulberry extract, whose value is 49.64 µg/mL.

## 4. Discussion

There is a growing interest in the beneficial effects of nutritional antioxidants on health through delaying ageing and age-related diseases [45]. Neuroprotection may be a result of the antioxidant and anti-inflammatory properties of phenolic compounds found in black mulberry [46]. Therefore, here, it was investigated whether *M. nigra* extract can act as a neuroprotective and antioxidant agent.

Neuro-2a is a neuroblast cell line derived from mice, used for its ability to produce microtubular proteins. There is no previous work carried out with black mulberry using this cell line, but it has been shown that it is not a cytotoxic extract at certain concentrations in the SF-295 neuronal line [47]. This research supports the findings regarding mitochondrial activity, as assessed by the MTT assay, indicating that the black mulberry extract exhibits no cytotoxicity within the physiological concentration range of 25–200 μg/mL.

To corroborate the data obtained, a toxicity test was accomplished in *Artemia salina*, *Metanauplius* (intermediate stage), and adults (where it reaches maturity). They are tiny crustaceans that live in inland lagoons and lakes; their body is thin and elongated [48]. In *Metanauplius,* the control remained at 70% survival, as well as at concentrations ranging from 25 to 400 μg/mL, while survival for the highest concentration (500 μg/mL) reduced viability to 45%. These results align with those previously obtained in the neuronal model, where the same high concentration tested resulted in decreased cell viability. In the adult stage, the control survival percentage reached 90%, and at every concentration tested, the percentage was 90% or above, stating greater resistance to the extract due to its ripening. After an extensive literature search, it seems to be the first test to evaluate toxicity in *M. nigra* within the scientific literature. As observed, the extract could be considered non-toxic to this crustacean because no significant differences were noted in the tested low concentrations compared to the control.

The generation of ROS is usually responsible for neuronal losses and is therefore related to neurodegenerative pathologies, such as Parkinson’s, Alzheimer’s, or Huntington’s disease. Oxidative damage comes from the oxidation of proteins, lipids, and DNA with ROS and free radicals [49]. Hence, it was decided to assess intracellular ROS production. This assay clearly demonstrates the antioxidant and neuroprotective activity of black mulberry extract in the N2a neuronal line. As depicted in Figure 3, when these cells were stimulated with hydrogen peroxide, production increased dramatically over the 90 min treatment period (160%). However, when co-treated (black mulberry and H_2_O_2_), fluorescence gradually decreased over the 10 measurements of the assay. Even at a concentration of 200 μg/mL, which initially started at very high levels of ROS, it decreased to levels comparable to those of the control cells at the final measurement, 82% and 85%, respectively. Compared to other studies involving neuronal treatment—HT22 cells from the mouse hippocampus—with myeloid beta oligomer (AβO) and a great amount of anthocyanins (100 mg/mL), these compounds manage to suppress ROS production [50]. In the hippocampus of adult mouse brains and in BV-2 microglial cells exposed to lipopolysaccharides, anthocyanin treatment causes LPS-induced ROS accumulation and oxidative stress to be attenuated [51]. Although these data cannot be extrapolated to neurons, they serve as a reference to observe the ROS-reducing potential of the anthocyanins in black mulberry extract. In the following assay with the DIV-7 neuronal cell line, the anthocyanin content at concentrations of 5 and 10 µg/mL demonstrated an ROS production of 70% and 60%, respectively, compared to 90% of the control [52], similar data to those of this study; the lowest doses (25, 50 and 100 μg/mL) decrease even below the control, which suggests that black mulberry extract could significantly suppress the accumulation of ROS, causing antioxidant enzymatic activity to be activated and mitochondrial function to be protected.

*C. elegans* has been chosen as an in vivo model to study the neuroprotection effect of *M. nigra* extract. As a model organism, *C. elegans* has several advantages, including the possibility of creating mutant strains to produce models to study specific diseases, such as the CL4176 strain for Alzheimer’s disease. This strain expresses human Aβ protein and has been shown to reproduce the key pathophysiology of Alzheimer’s disease: oxidative stress and fibril formation [53]. In this model, it was observed that nematodes treated with the extract exhibited delayed development of paralysis induced by the accumulation of Aβ protein (Figure 4). Our results were similar to those found by Wang et al. [54] for an aqueous extract of black mulberry fruits. Moreover, these authors reported a reduction in Aβ accumulation and suggested that *M. nigra* mitigates Alzheimer’s disease by activating the DAF-16 insulin signaling pathway, which is involved in the oxidative stress response, in *C. elegans*. Therefore, these findings support the neuroprotective potential related to the antioxidant activity of *M. nigra* extract.

After demonstrating the potential of this plant, an in vitro test was conducted targeting the central nervous system enzyme known as MAO-A. Inhibition of this pharmacological target can potentially lead to neuroprotective effects. MAO-A is an enzyme found in mitochondria that drives the oxidative deamination of some monoamines, such as serotonin, dopamine, adrenaline, and norepinephrine, which are important to maintain a normal mental state [55]. Regarding our study, extraordinary IC_50_ values of MAO-A were obtained. This suggests a substantial enzyme inhibition percentage of 49.64 µg/mL by the black mulberry extract, in comparison to 0.023 µg/mL inhibition by Clorgyline. The extract was able to inhibit the enzyme as well as the reference inhibitor in its entirety. Recent studies with anthocyanidins, anthocyanidins-3-glucosides, and anthocyanidins-3,5-diglucosides reported IC_50_ values of 29.2, 36.9, and 97.4 µM, respectively, indicating strong MAO-A inhibition, as well as very favourable changes in central nervous parameters of oxidative stress following anthocyanin administration [56].

Phenolic compounds, as oxygen radical scavengers, are generally related to antioxidant activity [57]. It has been studied that black mulberries are rich in polyphenols, acquiring the ability to inhibit lipid-soluble antioxidants [58]. The content of these polyphenols varies depending on the stage of fruit ripening. The black mulberry contains more phenolic compounds than the red mulberry, due to their difference in ripening [59].

As a complement to the investigation of ROS neutralization, the antioxidant profile was evaluated through the FRAP test to determine the total reducing capacity, obtaining brilliant results (12.48 µmol Fe^2+^/g.) in comparison with other similar studies which reflected results of 77.89 mg/g FeSO_4_ or 0.512 µmol Fe^2+^/g in terms of molarity [60], which is more than twenty times less than the extract studied in this project.

The imbalance between the antioxidant defence system and the production of ROS in living beings leads to the breakdown of cellular function and, therefore, oxidative damage. Inhibition of MAO-A prevents this damage, thus protecting mitochondrial function and acting as a neuroprotector. *M. nigra* extract has been shown to have both antioxidant and neuroprotective capacities, being a possible agent for the prevention of neurodegenerative diseases, thanks to its phenolic compounds, specifically anthocyanins, to which we attribute the modulation of the cellular antioxidant response.

In conclusion, black mulberry extract demonstrated the ability to reduce the intracellular ROS production induced by hydrogen peroxide and, therefore, exert a neuroprotective effect on this cell line. Furthermore, antiradical activity was demonstrated thanks to the in vitro FRAP assay, confirming the antioxidant potential of *M. nigra*.

## Data Availability

The original contributions presented in the study are included in the article, further inquiries can be directed to the corresponding author.
